# Poor oral function is associated with loss of independence or death in functionally independent older adults

**DOI:** 10.1371/journal.pone.0253559

**Published:** 2021-06-24

**Authors:** Yusuke Nagamine, Tsukasa Kamitani, Hajime Yamazaki, Yusuke Ogawa, Shunichi Fukuhara, Yosuke Yamamoto

**Affiliations:** 1 Department of Healthcare Epidemiology, School of Public Health in the Graduate School of Medicine, Kyoto University, Kyoto, Japan; 2 Department of Anesthesiology and Critical Care Medicine, School of Medicine, Yokohama City University, Yokohama, Japan; 3 Section of Clinical Epidemiology, Department of Community Medicine, Graduate School of Medicine, Kyoto University, Kyoto, Japan; University of Wisconsin Madison, UNITED STATES

## Abstract

**Aim:**

To clarify the association of poor oral function with loss of independence (LOI) or death in functionally independent older adults in the community.

**Methods:**

We conducted a secondary analysis of data from a prospective cohort study in two municipalities in Japan. We included participants who were older than 65 years of age and had no certification in long-term care at baseline. Poor oral function was evaluated by the Kihon Checklist. Among participants with poor oral function, they were further classified by the degree of quality of life (QOL) impairment due to dysphagia. Main outcome is LOI or death from all cause. The hazard ratio (HR) and 95% confidence of intervals (CIs) were estimated by Cox proportional hazard models adjusted for potential confounders.

**Results:**

Of 1,272 participants, 150 participants (11.8%) had poor oral function. The overall incidence of LOI or death was 10.0% in the participants with poor oral function, while 3.3% in the participants without. Participants with poor oral function were more likely to develop LOI or death than those without (crude HR = 3.17 [95% CIs 1.74–5.78], adjusted HR = 2.30 [95% CIs 1.22–4.36]). 10 participants (0.79%) were classified as poor oral function with QOL impairment, and were more likely to develop LOI or death than those without poor oral function (crude HR = 7.45 [95% CIs 1.80–30.91], adjusted HR = 8.49 [95% CIs 1.88–38.34]).

**Conclusions:**

Poor oral function was associated with higher risk of LOI or death in functionally independent older adults in the community.

## Introduction

Poor oral health, a major concern in geriatrics [1, 2], is a condition that is prevalent in the elderly and has adverse effects related to mastication and nutritional problems [3, 4]. Poor oral health is also strongly associated with malnutrition in the elderly, which can cause systemic muscle weakness and sarcopenia [1]. Oral uncleanness and oropharyngeal dysphagia, which are components of poor oral health, are important factors that can cause aspiration pneumonia [5]. Preventing poor oral health is critical for maintaining the health of older adults.

A longitudinal association between poor oral health and the progression of frailty has been reported in the elderly. Dentition status [6, 7] and maximum bite force [8] were associated with incidence of frailty. Some studies have investigated the association between poor oral health and long-term mortality. Tanaka *et al*. reported that “oral frailty” (evaluated by dental staff as 16 oral status) was significantly associated with the onset of disability and mortality [9]. Other studies reported that maximum occlusal force was associated with all-cause mortality in the elderly [10–12]. These studies, however, have relied on specialists to evaluate the condition of oral health. Fewer studies have been conducted to examine whether the assessment of poor oral health using self-administered questionnaires is associated with future outcomes.

The Kihon Checklist is a self-administered questionnaire, which was developed to simply screen older adults at high risk of needing nursing care [13, 14]. The Kihon Checklist includes three items to evaluate oral function, which can be separately used as a three-items questionnaire. However, the association of oral function evaluated by the Kihon Checklist with long-term prognosis has not been elucidated. The results of previous studies [15, 16] using the items of oral function in the Kihon Checklist have not shown a substantial relationship between the items of oral function and the need for nursing care and death for older adults. There are some notable limitations, such as a relatively short observation period and unadjusted variables (co-morbidities and motor function of the study participants). Further research is needed to fully understand these effects.

The purpose of this study is to clarify the association of poor oral function evaluated by the Kihon Checklist with loss of independence or death in functionally independent older adults in the community.

## Materials and methods

### Design and setting

We conducted a secondary analysis of data from the Locomotive Syndrome and Health Outcome in Aizu Cohort Study (LOHAS); a prospective cohort study in two municipalities (Tadami-cho and Minamiaizu-cho), in Fukushima prefecture, Japan. The study protocol of LOHAS has been published [17]. This prospective cohort study was started with the cooperation of the municipal governments of Tadami-cho and Minamiaizu-cho.

### Participants

In this cohort, we enrolled participants in an annual health checkup conducted in two municipalities. The annual health checkup was conducted by general physicians and provided by the municipal governments. We included participants who were enrolled in 2010, older than 65 years of age, and had no certification in long-term care insurance (LTCI) at baseline. We excluded participants who had missing data for exposure.

Japan’s LTCI system introduced in 2000 is a mandatory and universal entitlement for every Japanese person older than 65 years of age based strictly on physical and mental status [18]. LTCI care needs level (from 1 to 5) is determined by the nursing care needs certification board consisting of physicians, nurses, and other experts in health and social services [18]. When certified as needing nursing care by LTCI, a person can receive nursing care services with public insurance assistance. People certified at levels 3 or more are completely dependent on assistance for many activities of daily living [15]. LTCI certification status was obtained from the local government.

### Exposure

Poor oral function was defined as presenting two or more positive response to three questions on oral function in the Kihon Checklist [13, 14] (Ministry of Health, Labor and Welfare, Japan): (1) “Do you have any difficulties eating tough foods compared to 6 months ago?” (2) “Have you choked on your tea or soup recently?” (3) “Do you often experience having a dry mouth?”

Participants with poor oral function were further classified as to whether they had quality of life (QOL) impairment due to dysphagia using the following question: “Are you disturbed with your work or other activities due to difficulty in swallowing?” The question was answered by five-point scale: (1) no problem at all, (2) somewhat problematic, (3) problematic, (4) quite problematic, (5) serious problematic. Participants with a rating of ≥2 were classified as having QOL impairment due to dysphagia.

### Outcome

Primary outcome was loss of independence or death from all cause. Loss of independence was defined as developing new certification in LTCI needs level 3 or more. Data on death dates were obtained from death certificates with permission from the municipal governments.

### Covariates

We collected participants’ data including age, sex, body mass index (BMI), cerebrovascular disease, cognitive dysfunction, domain scores of physical functioning and mental health of 12-item short-form (SF-12) [19]. Scores of physical functioning and mental health were expressed as norm-based scores. Cerebrovascular disease was investigated by a self-administered questionnaire about history of cerebral infarction and cerebral hemorrhage. Cognitive dysfunction was defined as presenting one or more positive response to three questions on cognitive functions in the Kihon Checklist [13]: (1) “Do your family or your friends point out your memory loss?” (For example, “You ask the same question over and over again.”) (2) “Do you make a call by looking up phone numbers?” (3) “Do you find yourself not knowing today’s date?”

### Statistical analysis

Survival analysis was performed in participants without missing in covariates. Event was defined as incidence of loss of independence or death. The entry date of each participants was used as the starting point for survival analysis. Participants were followed up to March 31, 2014. Cox proportional hazard model was used to estimate hazard ratio (HR) and 95% confidential intervals (95% CIs). We calculated HR adjusted by potential confounders (age, male, BMI, cerebrovascular disease, cognitive dysfunction, SF-12 physical functioning, and SF-12 mental health). BMI was adjusted for categorical variables with normal range (18.5 to 24.9) as the reference. In the primary analysis, we analyzed the association of poor oral function with outcome. In the secondary analysis, we analyzed the association of poor oral function classified as having QOL impairment due to dysphagia with outcome.

To consider the effect of selection bias due to missing covariates in the population, we conducted the sensitivity analysis. We performed a multiple imputation procedure using the chained equation method based on the missing-at-random assumption for participants with missing one or more covariates. The missing values were imputed using poor oral function, incidence of loss of independence or death, and covariates. We created 20 imputed datasets, followed by integrated into one data set. Cox proportional hazard model was used to estimate HR and 95% CIs for the integrated dataset using the multiple imputation procedure.

All analyses were conducted using Stata version 15.1 (StataCorp, Texas, USA). A P value <0.05 (two-tailed) was set at statistically significant.

### Ethical considerations

This study was approved by the Research Ethics Committee of Kyoto University Graduate School of Medicine (R1730-1) and Fukushima Medical University (673), and all participants provided written informed consent.

## Results

**Fig 1:**
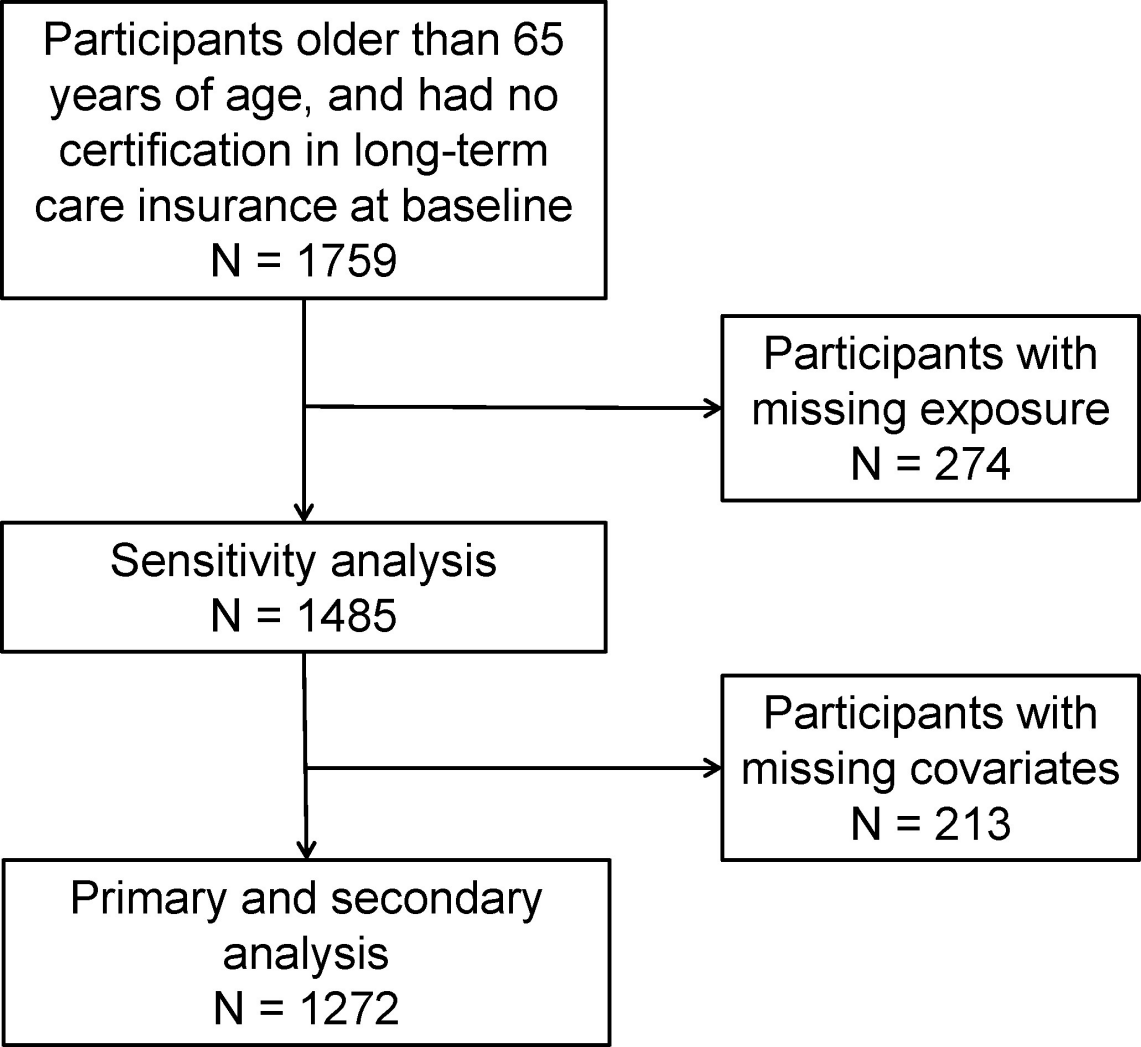
Flow chart of the study participants.

Fig 1 shows a flow chart of study participants. In total, 1759 participants who were older than 65 years of age and had no certification in LTCI were enrolled into the study in 2010. Among them, 274 participants (15.6%) had missing exposure data. Following exclusion of 213 participants who had missing at least one covariate variable, 1272 participants were included in the primary and secondary analyses. Exposure and covariates were collected using self-questionnaires, and some data were missing. Of these 1272 participants, 150 participants (11.8%) had poor oral function.

Table 1 shows the baseline characteristics of the participants included in the primary and secondary analysis. The mean age of the participants was 73.7 years (standard deviation (SD), 4.6), male was 43.1% of the participants. The average of BMI of the participants was 23.8 (SD, 3.0) kg/m^2^, 66 participants (5.2%) had cerebrovascular disease, and 421 participants (33.1%) had cognitive dysfunction. The average score of SF-12 physical functioning was 44.3 (SD, 14.1), and average score of SF-12 mental health was 49.6 (SD, 10.7) in the participants.

**Table 1:** Baseline characteristics of the study participants.

Characteristics	Total (N = 1272)	Poor oral function (-) (N = 1122, 88.2%)	Poor oral function (+) (N = 150, 11.8%)
Age, mean (SD), y	73.7 (4.6)	73.5 (4.6)	75.5 (4.4)
Male, N (%)	548 (43.1)	494 (44.0)	54 (36.0)
BMI, mean (SD), kg/m^2^	23.8 (3.0)	23.9 (2.9)	23.3 (3.0)
BMI, N (%)			
<18.5 kg/m^2^	30 (2.4)	22 (2.0)	8 (5.3)
18.5–24.9 kg/m^2^	812 (63.8)	714 (63.6)	98 (65.3)
≥25.0 kg/m^2^	430 (33.8)	386 (34.4)	44 (29.3)
Cerebrovascular disease, N (%)	66 (5.2)	56 (5.0)	10 (6.7)
Cognitive dysfunction, N (%)	421 (33.1)	335 (29.9)	86 (57.3)
SF-12 physical functioning, mean (SD) ^†^	44.3 (14.1)	45.0 (13.7)	39.5 (15.9)
SF-12 mental health, mean (SD) ^†^	49.6 (10.7)	50.0 (10.7)	47.1 (10.3)

*Notes*: SD = standard deviation; BMI = body mass index; SF-12 = Short Form-12

^†^Expressed as norm-based score

### Primary analysis

**Fig 2:**
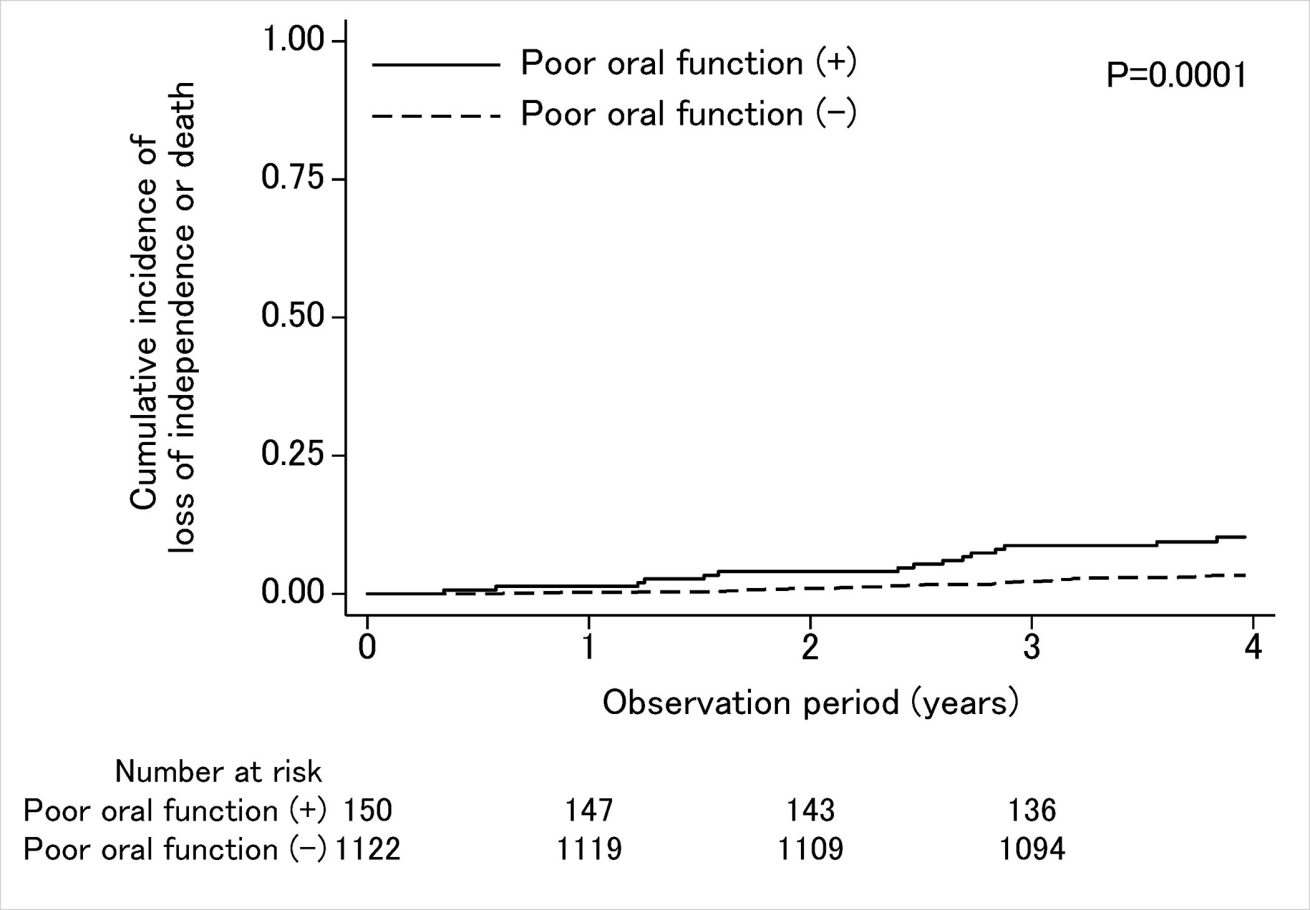
Kaplan–Meier curves of cumulative incidence of loss of independence or death according to the presence of poor oral function.

The median observation period was 1409 days. During the observation period, five participants (0.39%) censored due to relocation. The outcomes of the other participants were followed up until the end of the observation period. Fig 2 shows the Kaplan–Meier curves with outcomes of loss of independence or death depending on the presence of poor oral function. The result of the log-rank test was statistically significant (P = 0.0001).

The overall incidence of loss of independence or death was 3.3% (incidence rate, 0.0087/year) in those without poor oral function, while overall incidence was 10.0% (incidence rate, 0.0272/year) in those with poor oral function (Table 2).

**Table 2:** Association between poor oral function and loss of independence or death.

Oral function	Participants, N	Loss of independence or death, N (%)	Incidence rate (per year)	Crude HR (95% CIs)	Adjusted HR (95% CIs)
Poor oral function (-)	1122	37 (3.3)	0.0087	ref.	ref.
Poor oral function (+)	150	15 (10.0)	0.0272	3.17 (1.74–5.78)	2.30 (1.22–4.36)

*Notes*: Cox regression analysis was conducted with adjustments for age, male, body mass index, cerebrovascular disease, cognitive dysfunction, SF-12 physical functioning, and SF-12 mental health

HR = hazard ratio; CIs = confidence intervals

Participants with poor oral function were more likely to develop loss of independence or death compared than those without [crude HR = 3.17 (95% CIs 1.74–5.78), adjusted HR = 2.30 (95% CIs 1.22–4.36)].

### Secondary analysis

Ten participants (0.79%) were classified as having poor oral function with QOL impairment due to dysphagia. The overall incidence of loss of independence or death was 9.3% (incidence rate, 0.0251/year) in the poor oral function participants without QOL impairment due to dysphagia. Overall incidence was 20.0% (incidence rate was 0.0632/year) in the poor oral function participants with QOL impairment due to dysphagia (Table 3).

**Table 3:** Association between poor oral function with QOL impairment due to dysphagia and loss of independence or death.

Oral function	Participants, N	Loss of independence or death, N (%)	Incidence rate (per year)	Crude HR (95% CIs)	Adjusted HR (95% CIs)
Poor oral function (-)	1122	37 (3.3)	0.0087	ref.	ref.
Poor oral function (+)					
QOL impairment (-)	140	13 (9.3)	0.0251	2.92 (1.55–5.48)	2.09 (1.07–4.08)
QOL impairment (+)	10	2 (20.0)	0.0632	7.45 (1.80–30.91)	8.49 (1.88–38.34)

*Notes*: Cox regression analysis was conducted with adjustments for age, male, body mass index, cerebrovascular disease, cognitive dysfunction, SF-12 physical functioning, and SF-12 mental health

QOL = quality of life; HR = hazard ratio; CIs = confidence intervals

Participants who had poor oral function with QOL impairment due to dysphagia were likely to develop loss of independence or death than those without poor oral function [crude HR = 7.45 (95% CIs 1.80–30.91), adjusted HR = 8.49 (95% CIs 1.88–38.34)].

### Sensitivity analysis

S1 Table shows the characteristics of participants with and without missing covariates. In total 1485 participants who were imputed missing covariates, the overall incidence of loss of independence or death was 3.5% (incidence rate, 0.0092/year) in the participants without poor oral function, while overall incidence was 9.3% (incidence rate, 0.0253/year) in the participants with poor oral function.

Participants with poor oral function were more likely to develop loss of independence or death than those without [crude HR = 2.78 (95% CIs 1.55–4.98), adjusted HR = 2.03 (95% CIs 1.10–3.75] (S2 Table).

## Discussion

In our population-based cohort study, poor oral function evaluated by the Kihon Checklist was associated with higher risk of loss of independence or death in functionally independent older adults living in the community. Further, poor oral function with QOL impairment due to dysphagia was associated with a higher risk of loss of independence or death. This study suggests that poor oral function evaluated by the Kihon Checklist and QOL impairment due to dysphagia are risk factors for LTCI certification and death in older adults.

Assessment and evaluation of oral health are important considerations in older adults. Initial oral health assessment is recommended using the Oral Health Assessment Tool (OHAT) [20] which have 8 categories: lips, tongue, gums and tissues, saliva, natural teeth, dentures, oral cleanliness, and dental pain. However, this assessment tool does not include other oral function, for example, occlusal force, chewing, and swallowing. In recent years, oral hypofunction in older adults was defined by the Japanese Society of Gerodontology as the presentation of seven oral signs or symptoms: oral uncleanness, oral dryness, decline in occlusal force, decline in motor function of tongue and lips, decline in tongue pressure, decline in chewing function, and decline in swallowing function [21]. The diagnostic criteria for oral hypofunction are met when individuals express three or more out of seven items proposed [21]. By defining the concept and diagnosis of oral hypofunction, it is expected that epidemiological research will make further advancements in this field.

Evaluation of oral function by the Kihon Checklist is considered to evaluate decline in three items of oral symptoms subjectively. The question “Do you have any difficulties eating tough foods compared to 6 months ago?” measures decline in occlusal force. The question “Have you choked on your tea or soup recently?” measures decline in swallowing. The question “Do you often experience having a dry mouth?” measures oral dryness. It is advantageous that evaluation of oral function by the Kihon Checklist can be performed easily without examination by a medical or dental professional. In the future, easy assessment of oral function by the Kihon Checklist, which is associated with routine oral healthcare, may be applied in primary geriatric care.

In this study, we performed a detailed analysis by classifying participants with poor oral function according to the presence or absence of QOL impairment due to oropharyngeal dysphagia. We applied QOL impairment about swallowing as severity of oral function. In order to measure QOL for swallowing, SWAL-QOL is the most widely used tool [22]. SWAL-QOL is composed of 10 subscales, one of which is the burden related to swallowing. We believe that the question “Are you disturbed with your work or other activities due to dysphagia?” used to evaluate QOL impairment in this study was similar to the burden associated with swallowing. There are few studies showing the association between details of QOL related to swallowing function and loss of independence or death; however, this study can potentially be extended to include not only oral functions but also pharyngeal and swallowing functions.

Unlike the previous reports [15, 16], this study demonstrated the association between poor oral function evaluated by the Kihon Checklist and loss of independence or death. In the cohort study of Kabayama *et al*. [15], loss of independence was observed as an outcome, but death cases were excluded from the participants and could not be observed. In the cohort study of Kabayama *et al*. [15] and Okabe *et al*. [16], the observation period was about 3 years. This study differs from the previous studies in the following points. First, although our sample size is smaller, the median follow-up period of participants was longer (1409 days, approximately 4 years) than the previous studies. Second, only five cases (0.39%) were censored due to relocation, and participants’ outcome follow-up rates were high. Third, we adjusted proper potential confounders compared with the previous studies, which were BMI, cerebrovascular disease, cognitive dysfunction, and domain scores of physical functioning and mental health of SF-12.

The strength of this study was obtaining accurate information on LTCI certification and death with the cooperation of local governments. Further, detailed baseline data enabled us to make adjustments for potential confounders, including comorbidities, physical functioning, and mental health.

This study has several limitations. First, the subjects of this study were older adults living in rural areas, and not living in urban areas. It is considered that many participants were older adults engaged in agricultural work. Consequently, their daily physical functions were likely to be higher than those working in urban areas. The number of frail older adults may be relatively small. Therefore, the association of poor oral function with loss of independence or death may have been underestimated. Second, there have been no reports examining the validity of the questions on oral function in the Kihon Checklist with each symptom of oral hypofunction. Further research is needed on screening for oral hypofunction using the Kihon Checklist. Third, pharyngeal fiber optic examination, swallowing imaging, Volume-Viscosity Swallow Test (V-VST) [23], and other tests were not performed when evaluating oral function, diagnosing oropharyngeal dysphagia, or assessing severity. Although we used QOL impairment due to dysphagia as a measure of severity, the number of participants with QOL impairment due to dysphagia was as small (N = 10). In the future, it will be necessary to classify the severity of oropharyngeal dysphagia in more detail and to verify its association with outcomes. Fourth, there was the potential for selection bias owing to missing baseline data. The group with missing covariates tended to have a lower proportion of men, less cerebrovascular disease, and less cognitive dysfunction (S1 Table). We performed a sensitivity analysis with multiple imputations of missing covariates, and the results were similar to those of the primary analysis. However, the influence of selection bias owing to missing exposures cannot be ignored. Finally, there was the possibility of unmeasured confounders. In this study, adjustments were not made for the presence of neurodegenerative diseases (such as Parkinson’s disease) and malignant tumors. Although cognitive dysfunction was included as a potential confounder, its diagnosis was not always definitive and sufficient adjustments may not have been made.

## Conclusions

Poor oral function evaluated by the Kihon Checklist was associated with a higher risk of loss of independence or death in functionally independent older adults living in the community. Poor oral function with QOL impairment due to dysphagia was also associated with a higher risk of loss of independence or death. This study suggests the importance of public health in preventing poor oral function in older adults living in the community.

## Supporting information

S1 Table Participants characteristics with and without missing covariates. (DOCX)

S2 Table Association between poor oral function and loss of independence or death using multiple imputation-based sensitivity analysis. (DOCX)
