# Supplementary material for: Poor oral function is associated with loss of independence or death in functionally independent older adults
Source: PLoS One. 2021 Jun 24;16(6):e0253559. doi: 10.1371/journal.pone.0253559 (PMC8224883; doi:10.1371/journal.pone.0253559)
Supplement: S1 Table — (DOCX) [file pone.0253559.s001.docx]

**S1 Table. Participants characteristics with and without missing covariates**

| Characteristics | Missing covariates (-) (N = 1272) | Missing covariates (+) (N = 213) | Missing, N |
| --- | --- | --- | --- |
| Age, mean (SD), y | 73.7 (4.6) | 74.8 (4.2) | 0 |
| Male, N (%) | 548 (43.1) | 73 (34.3) | 0 |
| BMI, mean (SD), kg/m^2^ | 23.8 (3.0) | 24.4 (3.3) | 174 |
| BMI, N (%) |  |  |  |
| <18.5 kg/m^2^ | 30 (2.4) | 0 (0.0) | ‒ |
| 18.5 ‒ 24.9 kg/m^2^ | 812 (63.8) | 25 (64.1) | ‒ |
| ≥25.0 kg/m^2^ | 430 (33.8) | 14 (35.9) | ‒ |
| Cerebrovascular disease, N (%) | 66 (5.2) | 6 (3.8) | 56 |
| Cognitive dysfunction, N (%) | 421 (33.1) | 44 (24.9) | 36 |
| SF-12 physical functioning, mean (SD) ^†^ | 44.3 (14.1) | 42.4 (14.7) | 1 |
| SF-12 mental health, mean (SD) ^†^ | 49.6 (10.7) | 50.7 (10.0) | 7 |
| Loss of independence or death, N (%) | 52 (4.1) | 9 (4.2) | 0 |
| Death, N (%) | 30 (2.4) | 8 (3.8) | 0 |
| *Notes:* SD = standard deviation; BMI = body mass index; SF-12 = Short Form-12  ^†^ Expressed as norm-based score | | | |
