# Supplementary material for: Poor oral function is associated with loss of independence or death in functionally independent older adults
Source: PLoS One. 2021 Jun 24;16(6):e0253559. doi: 10.1371/journal.pone.0253559 (PMC8224883; doi:10.1371/journal.pone.0253559)
Supplement: S2 Table — (DOCX) [file pone.0253559.s002.docx]

**S2 Table. Association between poor oral function and loss of independence or death using multiple imputation-based sensitivity analysis**

| Oral function | Participants, N | Loss of independence or death, N (%) | Incidence rate (per year) | Crude HR (95% CIs) | Adjusted HR (95% CIs) |
| --- | --- | --- | --- | --- | --- |
| Poor oral function (-) | 1323 | 46 (3.5) | 0.0092 | ref. | ref. |
| Poor oral function (+) | 162 | 15 (9.3) | 0.0253 | 2.78 (1.55 ‒ 4.98) | 2.03 (1.10 ‒ 3.75) |
| *Notes:* Cox regression analysis was conducted with adjustments for age, male, body mass index, cerebrovascular disease, cognitive dysfunction, SF-12 physical functioning, and SF-12 mental health | | | | | |
| HR = hazard ratio; CIs = confidence intervals | | | | | |
